# Concurrent epigenetic silencing of wnt/β-catenin pathway inhibitor genes in B cell chronic lymphocytic leukaemia

**DOI:** 10.1186/1471-2407-12-213

**Published:** 2012-06-06

**Authors:** Evgeny A Moskalev, Katrin Luckert, Ivan A Vorobjev, Sergey E Mastitsky, Aleena A Gladkikh, Achim Stephan, Marita Schrenk, Kamil D Kaplanov, Olga B Kalashnikova, Oliver Pötz, Thomas O Joos, Jörg D Hoheisel

**Affiliations:** 1 Functional Genome Analysis, Deutsches Krebsforschungszentrum (DKFZ), Im Neuenheimer Feld 580, 69120, Heidelberg, Germany; 2 Biochemistry Department, NMI Natural and Medical Sciences Institute at the University of Tübingen, Markwiesenstr. 55, 72770, Reutlingen, Germany; 3 Functional Morphology of Hemablastoses, National Hematology Research Centre of Russian Academy of Medical Sciences, Novy Zykovsky passage 4a, 125167, Moscow, Russia; 4 A.N. Belozersky Institute and Biological Faculty, Moscow State University, Leninskie Gory 1, 119991, Moscow, Russia; 5 Theoretical Bioinformatics, Deutsches Krebsforschungszentrum (DKFZ), Im Neuenheimer Feld 580, 69120, Heidelberg, Germany; 6 Department of Haematology, Volgograd Regional Clinical Oncological Dispensary No.1, Zemlyachki str. 78, 400138, Volgograd, Russia; 7 Diagnostic Molecular Pathology, Institute of Pathology, University of Erlangen-Nürnberg, Krankenhausstr. 8-10, 91054, Erlangen, Germany

**Keywords:** B cell chronic lymphocytic leukaemia, Wnt/β-catenin pathway, Inhibitor genes, DNA hypermethylation, Epigenetic silencing, β-catenin

## Abstract

**Background:**

The Wnt/β-catenin signalling is aberrantly activated in primary B cell chronic lymphocytic leukaemia (CLL). Epigenetic silencing of pathway inhibitor genes may be a mechanism for its activation. In this study, we investigated systematically and quantitatively the methylation status of 12 Wnt/β-catenin pathway inhibitor genes – *CDH1, DACT1, DKK1, DKK2, DKK3, DKK4, SFRP1, SFRP2, SFRP3, SFRP4, SFRP5* and *WIF1* – in the cell lines EHEB and MEC-1 as well as patient samples.

**Methods:**

Quantification of DNA methylation was performed by means of bisulphite pyrosequencing and confirmed by bisulphite Sanger sequencing. Gene expression was analysed by qPCR using *GAPDH* as internal control. E-cadherin and β-catenin protein quantification was carried out by microsphere-based immunoassays. Methylation differences observed between the patient and control groups were tested using generalised least squares models.

**Results:**

For 10 genes, a higher methylation level was observed in tumour material. Only *DKK4* exhibited similarly high methylation levels in both tumour and normal specimens, while *DACT1* was always essentially unmethylated. However, also for these inhibitors, treatment of cells with the demethylating agent 5-aza-2´-deoxycytidine resulted in an induction of their expression, as shown by quantitative PCR, suggesting an indirect epigenetic control of activity. While the degree of demethylation and its transcriptional consequences differed between the genes, there was an overall high correlation of demethylation and increased activity. Protein expression studies revealed that no constitutive Wnt/β-catenin signalling occurred in the cell lines, which is in discrepancy with results from primary CLL. However, treatment with 5-aza-2´-deoxycytidine caused accumulation of β-catenin. Simultaneously, E-cadherin expression was strongly induced, leading to the formation of a complex with β-catenin and thus demonstrating its epigenetically regulated inhibition effect.

**Conclusions:**

The results suggest an epigenetic silencing mechanism of the Wnt/β-catenin pathway inhibitor genes in CLL. Hypermethylation and silencing of functionally related genes may not be completely stochastic but result from the tumour epigenome reprogramming orchestrated by Polycomb-group repressive complexes. The data are of interest in the context of epigenetic-based therapy.

## Background

Constitutive Wnt/β-catenin signalling is increasingly recognised to be a vital ingredient for the malignant phenotype of different types of tumours including the most common haematological malignancy, B cell chronic lymphocytic leukaemia (CLL) [1-4]. The Wnt pathway is indispensable for normal embryonic development and cell differentiation, including that of the B cell lineage [5]. Being dormant in normal peripheral B-lymphocytes [3], the Wnt pathway is aberrantly activated in CLL and contributes substantially to the anti-apoptotic and mitogenic characteristics of CLL B cells [1,3,6]. Inhibition of key pathway components suppresses survival of CLL B cells *ex vivo* and in a xenograft model [1,6,7]. Therefore, the mechanisms underlying aberrant functioning of the Wnt pathway are of considerable therapeutic interest. In addition, the recent finding of active Wnt/β-catenin signalling in the pre-leukemic state of monoclonal B cell lymphocytosis could suggest the potential of CLL prevention by targeting the pathway early during the development of CLL [3].

The Wnt pathway operates by stabilising the key downstream effector β-catenin in the cytoplasm [8]. In the non-activated state of the pathway, cytoplasmic β-catenin undergoes constant N-terminal phosphorylation at the residues S33, S37, T41 and S45, which act as covalent marks for proteasomal degradation [2]. Pathway activation occurs upon binding of the growth factors of the Wnt class to the membrane receptors of the Frizzled family (FZD) and prevents β-catenin from being degraded. As a consequence, its translocation to the nucleus is promoted, where it forms a transcriptionally active complex with the members of the T-cell factor/lymphocyte enhancer factor (TCF/LEF) family of transcription factors and induces the expression of pro-survival and proliferative genes (e.g., *cyclin Dc-myc*) [8]. Signal transduction is negatively controlled by multiple physiological inhibitors. These belong to several protein families and operate at different points along the pathway. Secreted frizzled-related proteins 1 to 5, DKK1 to 4 and WIF1 prevent the induction of signalling by interfering with the upstream components on the cell surface, namely the Frizzled receptor and the LRP5-6 co-receptor [8]; DACT1 induces degradation of the cytoplasmic effector Dishevelled [9]; the adhesion molecule E-cadherin (CDH1) directly binds β-catenin on the cellular membrane, thereby sequestering it from the cytoplasm [10].

The mechanism of the aberrant functioning of the Wnt/β-catenin pathway in CLL remains only incompletely understood [1]. Some data suggest that at least partially it may rely on the epigenetic silencing of pathway inhibitors. Aberrant hypermethylation of some antagonistically active genes has been reported as a mechanism for pathway induction in several types of solid tumours [11-13]. Additionally, for a variety of human malignancies, there is growing evidence that an epigenetic inactivation of the Wnt/β-catenin pathway inhibitors is associated with a tumour-favourable phenotypic outcome [14]. Little is known, however, about the functional relevance to CLL. The current knowledge is largely limited to the qualitative description of the methylation status of individual genes in patient material. Aberrant hypermethylation and its role in the loss of expression have been shown for some *SFRP* family members [15-18] but there are only fragmentary data about the methylation status of the other Wnt/β-catenin antagonists in CLL [15,17,19].

Using specialised oligonucleotide microarrays, we had identified aberrant promoter methylation of *DKK2* and *DKK3* and confirmed earlier findings for *SFRP1, SFRP2* and *SFRP4*[16] on a limited number of primary CLL samples (unpublished data). This led us to formulating the hypothesis that the different inhibitors of the Wnt/β-catenin pathway may undergo concordant aberrant hypermethylation in malignant B cells, thereby contributing to the development of CLL. In this study, we investigated systematically and quantitatively the methylation status and the expression of the genes of twelve Wnt/β-catenin pathway inhibitors in two CLL cell line models and primary CLL B cells. We found a strong association of hypermethylation and transcriptional regulation of the antagonists in CLL. In addition, protein analyses revealed that no constitutive expression of ß-catenin occurred in the cell lines, as opposed to results from primary CLL. However, treatment with 5-aza-2´-deoxycytidine restored β-catenin expression. As a confirmation that variation of methylation is not just directly regulating the expression of ß-catenin while the variations in the antagonist genes are coincidental but functionally irrelevant, we also studied the protein expression and functioning of the antagonist factor adhesion molecule E-cadherin (CDH1) upon pharmacologically induced DNA demethylation.

## Methods

### Cell culture, drug treatment and patient samples

The chronic lymphocytic leukaemia cell lines MEC-1 [20] and EHEB [21] were obtained from the German Collection of Microorganisms and Cell Cultures (DSMZ, Braunschweig, Germany) and were grown in a medium consisting of 90% Iscove's Modified Dulbecco's Medium (Invitrogen, San Diego, USA) or Roswell Park Memorial Institute Medium (Invitrogen), respectively, supplemented with 10% foetal bovine serum (Invitrogen). Treatment of cells with 5-aza-2´-deoxycytidine (5-aza-dC; Merck Chemicals, Nottingham, UK) was performed for 72 h and 96 h, starting cultures from 5 × 10^5^ cells/ml. For the 96 h-treatment, the medium was changed after 48 h in order to supply fresh drug. Since a treatment with 0.5 μM 5-aza-dC resulted in only a small decrease of methylation of hypermethylated genes (on average by 9.5%), further experiments were performed at higher drug concentrations (1.0 and 2.0 μM), which did not affect the per cent of viable cells in cultures (>90%) as shown with Vi-Cell Automated Cell Viability Analyzer (Beckman Coulter, Brea, USA). These 5-aza-dC concentrations, but not lower doses, proved to be effective in DNMT1 depletion and transcriptional reactivation of different genes in CLL cell line WaC3CD5 in an earlier study [22] as well as in other cell lines of B cell lineage [23,24].

Peripheral blood was obtained from 12 patients of Volgograd Regional Clinical Oncological Dispensary No.1 (Volgograd, Russia) and the National Haematology Research Centre of the Russian Academy of Medical Sciences (Moscow, Russia). Written informed consent was given and the experiments were approved by the institutional Ethical Review Boards. Diagnosis of CLL was established according to standard morphologic and immunophenotypic criteria [25]. All patients were untreated at the time of blood collection. Peripheral blood mononuclear cells were isolated by a standard procedure using Ficoll-Hypaque gradient centrifugation as described elsewhere [26]. A total of five buffy-coats from the peripheral blood of healthy individuals were provided by the Institute for Clinical Transfusion Medicine and Cell Therapy (Heidelberg, Germany). CD19^+^ cells were isolated from control samples using Dynabeads CD19 pan B (Invitrogen, Carlsbad, USA) according to the manufacturer’s protocol and employed as a reference. The cells were collected and snap-frozen for the subsequent extraction of nucleic acids. The patient data are summarised in Additional file 1: Table S 1.

### DNA isolation and bisulphite conversion

DNA was extracted from the samples using the QIAamp DNA Blood Mini Kit (Qiagen, Hilden, Germany) as suggested by the manufacturer. DNA concentration was measured in a ND-1000 spectrophotometer (Thermo Scientific, Wilmington, USA). A total of 1.9 μg DNA was treated with sodium bisulphite using the EpiTect Bisulfite kit (Qiagen). The efficiency of bisulphite conversion averaged 98.8% and was computed from the sequences of 231 cloned PCR-products of *CDH1, DACT1, DKK1, DKK2, DKK3, DKK4, SFRP1, SFRP2, SFRP3, SFRP4, SFRP5* and *WIF1* using the BISMA software, which considers the non-CpG cytosines within the sequences [27].

### PCR amplification

PCR-amplification of the loci interrogated was carried out in 25 μl reactions that contained 2.0 μl bisulphite-converted DNA, 1.5 mM MgCl_2_, 125 mM dNTP, 200 nM primers, 0.65 units HotStar Taq DNA polymerase and 1x Q-solution (Qiagen). A previously reported amplification protocol was employed [28]. Briefly, amplification was started by an initial activation of the HotStar Taq DNA polymerase at 95°C for 15 min. The first amplification cycle was denaturation at 95°C for 1 min, annealing at 62°C for 2 min and elongation at 72°C for 3 min. This procedure was continued for 20 cycles, reducing the annealing temperature by 0.5°C each cycle, followed by 25 cycles of 1 min denaturation at 95°C, 2 min annealing at 52°C and 2 min elongation at 72°C. The sequences of the PCR primers are listed in Table 1. About 5 μl of each reaction was examined on 2% agarose gels.

**Table 1:** Sequences of the PCR and pyrosequencing primers used in this study

**Gene symbol**	Primer sequences (5´-3´) * F: PCR forward; R: PCR reverse; **S: pyrosequencing; bio: biotinylation**	**Amplicon length, bp**	**Number of CpGs quantified by pyrosequencing**	**Reference**
CDH1-F	TTTTTTTTGATTTTAGGTTTTAGTGAG	421		[29]
CDH1-R	bio-ACTCCAAAAACCCATAACTAACC			
CDH1-S	AGTTAGTTTAGATTTTAGTT		9	this study
DACT1-F	GTTTGGGAAGTGAAAGAAATTTAATT	184		[30]
DACT1-R	bio-CTAAAACCCCAACATCCTATTACAAT			
DACT1-S	AGATTGTGTTGTAATTTGGT		5	this study
DKK1-F	bio-GGGGTGAAGAGTGTTAAAGGTT	326		[31]
DKK1-R	AAACCATCATCTCAAAAAAACTCAA			
DKK1-S	CTACAAAAAACACAAAACTCTAC		8	this study
DKK2-F	bio-TTTTAGTAGTTGTGGGTGGAGATA	456		this study
DKK2-R	ATACTCCTTTTCAAAATTAACAAAC			
DKK2-S	CCTAACTCACAAAAAACAAC		11	
DKK3-F	GATTTTGTTGAGTTTAGTTTTTTTTGGT	123		[32]
DKK3-R	bio-CAAACCTCTCTCAACCCCTACCTA			
DKK3-S	TTTTTTGGTGGATGTG		5	this study
DKK4-F	bio-ATAGATTTGAAGGGATTTGTTGAAGTTT	328		[33]
DKK4-R	CAAAACCAACTCAACCCCAACAAAAC			
DKK4-S	CTAAACTAACAACTCAACAC		2	this study
SFRP1-F	TTTTTAAGGGGTGTTGAGT	412		[16]
SFRP1-R	CAAACTTCCAAAAACCTCC			
SFRP1-S	GGAGTTGATTGGTTG (Sanger sequencing)			this study
SFRP2-F	ATGTTTGGTAATTTAGTAGAAATTT	409		this study
SFRP2-R	bio-CAACCAAAATTTTCTTAACCTTTTT			
SFRP2-S	GATTGGGGTAAAATAAGTT		14	
SFRP3-F	bio-GTGATTTAGGGGAGGAGATATTTTAGA	542		this study
SFRP3-R	TTCCAAAACAAAAACTTACACAAAA			
SFRP3-S	CAAAATAAAACAAAATACAAC		4	
SFRP4-F	bio-GTGTTTTGTGTGTTAGA	220		[16]
SFRP4-R	CCACTAAAATAAAAAAAAACATAACA			
SFRP4-S	TACCACCCTCATCTTTC		2	this study
SFRP5-F	GTAGGGAGTTTTGGGGAGAAA	272		[16]
SFRP5-R	bio-CCCAAATAAATAACAACCTAC			
SFRP5-S	GTTTTGGAGTTGGGGTTAG		8	this study
WIF1-F	bio-GAGTGATGTTTTAGGGGTTT	414		[34]
WIF1-R	CCTAAATACCAAAAAACCTAC			
WIF1-S	AAACTACATTCACAATAC		7	this study

* Primers of the same sequence but without biotin modification were employed for amplification of the PCR-products used for subcloning and bisulphite sequencing.

In order to control for possible amplification bias, appropriate calibration was performed as described in detail [28]. Fully methylated and unmethylated human control DNA that had been bisulphite-treated was purchased (EpiTect PCR control DNA; Qiagen) and mixed in different ratios to obtain calibration samples that represent distinct methylation percentages of 0, 12.5, 25, 37.5, 50, 62.5, 75, 87.5 and 100%, respectively. A total of 15 ng calibration DNA was used for the amplification of each locus.

### Bisulphite pyrosequencing

A volume of 20 μl of each PCR product was mixed with 2 μl Streptavidin Sepharose High Performance (GE Healthcare, Uppsala, Sweden), 38 μl PyroMark binding buffer (Qiagen) and 20 μl water. The Vacuum Prep Workstation (Biotage, Uppsala, Sweden) was used to prepare single-stranded DNA according to the manufacturer’s instructions. The Sepharose beads with the single-stranded templates attached were released into a PSQ 96 Plate Low (Biotage) containing 15 μl of 0.6 μM corresponding sequencing primer in annealing buffer. Pyrosequencing reactions were carried using the Pyro Gold Reagent Kit (Biotage) in a PSQ HS 96 Pyrosequencing System (Biotage) according to the manufacturer’s protocol. The sequences of the pyrosequencing primers are listed in Table 1. Quantification of CpG methylation was performed using the Software PyroQ-CpG v.1.0.9 (Biotage). The initial amplification result containing a bias towards unmethylated alleles was corrected using the calibration data derived from the control samples as previously described [28]. The PCR-product of *SFRP1* was sequenced directly using Sanger chemistry. Despite optimisation efforts, accurate quantification was not possible by alternative pyrosequencing assays, which resulted either in enormous bias towards the methylated allele or readouts with lack of specificity.

### Bisulphite Sanger sequencing

The PCR products were purified with the QIAquick PCR Purification kit (Qiagen) and cloned using the TOPO TA Cloning kit (Invitrogen). Clones were picked at random and sequenced with Sanger chemistry at GATC Biotech (Constance, Germany). The sequencing data were visualised using the CpGviewer software [35].

### Statistical analysis

For each locus, the average methylation percentage across the interrogated CpG sites was calculated. Differences observed between the patient and control groups were tested using generalised least squares (GLS) models [36]. As the country of origin (Russia or Germany), sex, and age of the probands might have been influential covariates, they were taken into account. Thus, we fitted the following model for each gene:

(1) $$\sqrt{\text{Methylation}}=\beta_{0}+\beta_{1}\text{Country}+\beta_{2}\text{Status}+\beta_{3}\text{Sex}+\beta_{4}\text{Age}+\varepsilon \text{,}$$

where $\sqrt{\text{Methylation}}$ are the methylation measurements that were square root-transformed to achieve normality; *β*_0 _is the model intercept; *β*_1 _is the effect of the country of origin, which is used as a binary variable that takes value 0 for Germany and 1 for Russia; *β*_2 _is the effect of the proband’s status (a binary variable 0 for healthy and 1 for a diseased individual); *β*_3 _is the effect of sex (0 for a female and 1 for a male individual); *β*_4 _is the effect of age, and *ε *represents the model residuals. Ideally, model residuals should be normally distributed with mean 0 and a certain constant variance. However, an exploratory analysis revealed high variation of methylation between the countries as well as between the healthy and diseased individuals. The homogeneity of variance assumption was thus relaxed by allowing the variance to be different in each stratum, i.e. in each of the combinations of country and disease status [36].

The full model was then reduced by stepwise backward elimination of insignificant terms. For each gene, only the final optimal model found by this approach is reported. Validation of the optimal models was performed by an examination of the quantile-quantile plots of their residuals [36]. Estimation of the model parameters was conducted based on the restricted maximum log-likelihood algorithm using the nlme v3.1-102 package [37] for the R computing environment [38]. *P*-values of less than 0.05 were regarded as statistically significant.

### RNA extraction and quantitative RT-PCR

Total RNA was isolated using the miRNeasy Mini Kit (Qiagen) as suggested by the manufacturer. The RNA concentration was measured in a ND-1000 spectrophotometer (Thermo Scientific). The quality of the RNA samples was confirmed in a 2100 Bioanalyzer (Agilent, Santa Clara, CA). The RNA integrity number [39] of the samples averaged 9.7. Reverse transcription reactions were carried out with 1 μg of total RNA using SuperScript III Reverse Transcriptase (Invitrogen) and 0.5 μg oligo(dT) 12–18 primer (Invitrogen). Quantitative RT-PCR was performed in triplicate with the ABI PRISM 7900 Sequence Detection System (ABI, Foster City, USA) using the Absolute QPCR SYBR Green mix (Thermo Scientific). The identity codes of the commercially available PCR-primers are as follows: Hs_CDH1_1_SG, Hs_DACT1_2_SG, Hs_DKK1_1_SG, Hs_DKK2_1_SG, Hs_DKK4_1_SG, Hs_SFRP1_1_SG, Hs_SFRP2_1_SG, Hs_FRZB_2_SG, Hs_SFRP4_3_SG, Hs_WIF-_1_SG, Hs_GAPDH_1_SG (Qiagen), DKK3-qRTF1 and DKK3-qRTR1 (Thermo Scientific). Primer sequences for *SFRP5* have been reported earlier: 5´-CTGACGGCCTCATGGAGCAGATGT-3´ (forward) and 5´-TCCAATCAGCTTCCGG TCCCCATT-3´ (reverse) [16]. Universal Human Reference (UHR) total RNA was used as a calibration control (Stratagene, La Jolla, USA). The calibration graph (c_t_ vs. log unit of the standard template) was obtained as described [40]. Two housekeeping genes – glyceraldehyde-3-phosphate dehydrogenase (*GAPDH*) and β-actin (*ACTB*) – were tested. *GAPDH* was superior over *ACTB* exhibiting only minor variation of expression in all studied conditions (CV 10.4% vs. 34.9%, respectively) and therefore served as an internal control. The thermal cycler conditions were as follows: 50°C for 2 min and 95°C for 15 min, followed by a two-step PCR of 45 cycles at 95°C for 15 sec and 60°C for 60 sec.

### Quantification of E-cadherin and β-catenin expression

For protein extraction, cells were lysed shaking at 4°C for 30 min in a buffer containing 50 mM Tris/HCl (pH 7.4), 150 mM NaCl, 1% Triton X-100, 1x Complete Protease Inhibitor (Roche Diagnostics, Mannheim, Germany), 1x phosphatase inhibitor I and III (Sigma-Aldrich, St. Louis, USA) and 2.5 U/ml benzonase (Qiagen). Cells were passed through a 25 gauge needle, and cell debris was removed by centrifugation at 15,000 g for 30 min at 4°C.

Protein quantification was performed by an immunoassay as previously described [41]. An antibody that is specific for the C-terminus of β-catenin (BD Biosciences, Franklin Lakes, USA) was covalently immobilised on magnetic xMAP microspheres (Luminex, Austin, Texas) [42]. Per well of a microtiter plate (non-binding surface; Corning, New York, USA), 2,000 beads in 20 μl blocking reagent for ELISA (Roche Diagnostics) were incubated with 20 μg protein from cell lysates in 40 μl with lysis buffer. In calibration experiments, defined amounts of recombinantly expressed GST-β-catenin were used. Incubation was overnight at 4°C. The samples were transferred to a blocked filter plate (Millipore, Billerica, USA) and washed twice with 100 μl PBS using a vacuum manifold (Millipore). For detection, 30 μl of anti total β-catenin antibody (Invitrogen; 1:200 diluted in the assay buffer) were added to the beads. Incubation was at room temperature for 120 min. Unbound antibody was removed by washing with PBS as described above. A volume of 30 μl of 2.5 μg/ml donkey anti-rabbit phycoerythrin-conjugated antibody (Jackson ImmunoResearch, West Grove, USA) was added next and incubated for 45 min at room temperature. After another washing with PBS, the beads were resuspended in 100 μl assay buffer and analysed with a Luminex 100 IS system (Luminex Corp, Austin, USA). Calculation of the absolute β-catenin expression was based on a seven-point dilution series of the recombinant standard protein.

For the quantification of E-cadherin, an E-cadherin-specific antibody (R&D Systems, Minneapolis, USA) was employed as the bead-bound capture reagent. All dilution, incubation, washing and measurement steps were performed as described above. However, the detection system consisted of biotinylated detection antibodies specific for E-cadherin (R&D Systems) and phycoerythrin-conjugated Leandro, USA). Recombinant human E-cadherin Fc Chimera (R&D Systems) was used as standard.

## Results

### Wnt/β-catenin pathway inhibitor genes are concurrently hypermethylated both in CLL cell lines and patient samples

**Figure 1:**
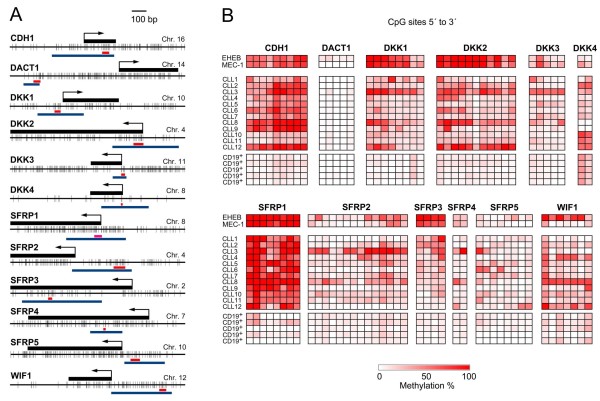
**Maps of the studied sequences. **(**A**) Schematic representations of the genomic regions are shown. The gene names and the chromosomal locations are given. Vertical bars indicate the positions of CpG dinucleotides. Exons are shown above as black rectangles; arrows indicate the known or presumed transcriptional start sites. Red bars below specify the regions analysed by bisulphite pyrosequencing; blues bars indicate the area studied with bisulphite Sanger sequencing; the magenta bar shows the region studied by direct bisulphite sequencing of the *SFRP1 *PCR-product. (**B**) Methylation patterns of the Wnt/β-catenin pathway inhibitor genes. Each square represents a CpG site. The degree of methylation was measured by bisulphite pyrosequencing in the EHEB and MEC-1 cell lines, in 12 patient samples (CLL1 to CLL12) and CD19^+^ B cells from healthy donors. At the bottom, an intensity scale is shown. Results for *SFRP1* were obtained by direct Sanger sequencing of the respective amplicon.

To find out if the expression of the Wnt/β-catenin pathway inhibitors may be regulated by DNA methylation, the two CLL cell lines EHEB and MEC-1 were studied. First, bisulphite pyrosequencing was performed to examine the methylation status of twelve inhibitor genes. After correction of the raw data for PCR-bias and artificial variations introduced by the pyrosequencing process as described [28], accurate quantification of the methylation degree was performed for CpG dinucleotides that are located in proximity to the transcriptional start sites or those associated with expression down-regulation of the respective genes in solid tumours [30,34] (Figure 1A). A high degree of methylation was recorded (average methylation percentage in EHEB/average methylation percentage in MEC-1) for *CDH1* (79/79), *DKK1* (68/75), *DKK2* (88/83), *SFRP3* (94/71) and *WIF1* (71 in EHEB). Partial methylation was observed for *DKK3* (23/37), *DKK4* (31 in EHEB), *SFRP2* (32/17) and *SFRP4* (24/29). The genes *DACT1* (10/0), *SFRP5* (14/6), *DKK4* (9 in MEC-1) and *WIF1* (11 in MEC-1) were essentially unmethylated. Pyrosequencing of *SFRP1* failed, although various primers were tested. However, extensive hypermethylation of *SFRP1* in both cell lines was confirmed by semiquantitative bisulphite Sanger sequencing. Overall, ten out of twelve antagonists of Wnt/β-catenin signalling exhibited substantial methylation of the gene in at least one of the two CLL cell line models.

Because DNA methylation in established cancer cell lines may not always adequately reflect the reality in primary tumours, also patient and control samples were studied for confirmation (Figure 1B). Consistent with the observations in the cell lines, eleven genes were found to be at least partially methylated in the patient material (average methylation percentage across twelve patient samples; lowest and highest value): *CDH1* (56; 22 to 88), *DKK1* (34; 17 to 84), *DKK2* (35; 14 to 78), *DKK3* (18; 5 to 61), *DKK4* (32; 0 to 71), *SFRP2* (21; 2 to 70), *SFRP3* (26; 6 to 50), *SFRP4* (17; 3 to 68), *SFRP5* (15; 6 to 27), *WIF-1* (30; 8 to 73). Again, extensive hypermethylation of *SFRP1* was shown by semiquantitative bisulphite Sanger sequencing. In contrast, and in accordance with the cell line data, *DACT1* was basically unmethylated (2; 1 to 5) in all samples. All the loci interrogated were essentially unmethylated in control CD19^+^ B cells of five healthy individuals with the exception of *DKK4*, which was found to be methylated (36; 33 to 39) to a level very similar to that in the cancer samples.

**Figure 2:**
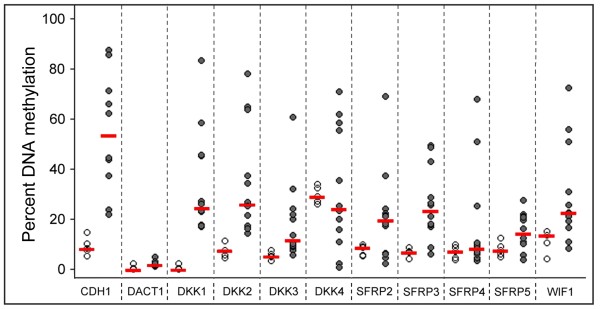
**Methylation of the inhibitor genes in patient CLL samples. **Methylation in tumour cells (filled circles) and control CD19^+^ B cells (empty circles) were analysed by bisulphite pyrosequencing. Each circle indicates the methylation degree of a particular sample. Horizontal bars denote the median methylation level for the patient group or the healthy donors, respectively.

The methylation patterns of most genes were heterogeneous within the patient group. However, the levels of methylation for *CDH1DKK1DKK2DKK3SFRP3* and *WIF1* were significantly higher than in the control group (Figure 2). Although there was no statistically significant difference between the patient and control groups for the other genes, abnormally high methylation – defined as an increase of the average methylation level beyond that of the average observed in the group of normal tissues plus twice the standard deviation [43] – was a frequent event in patient CLL samples but for *DACT1* and *DKK4*.

**Figure 3:**
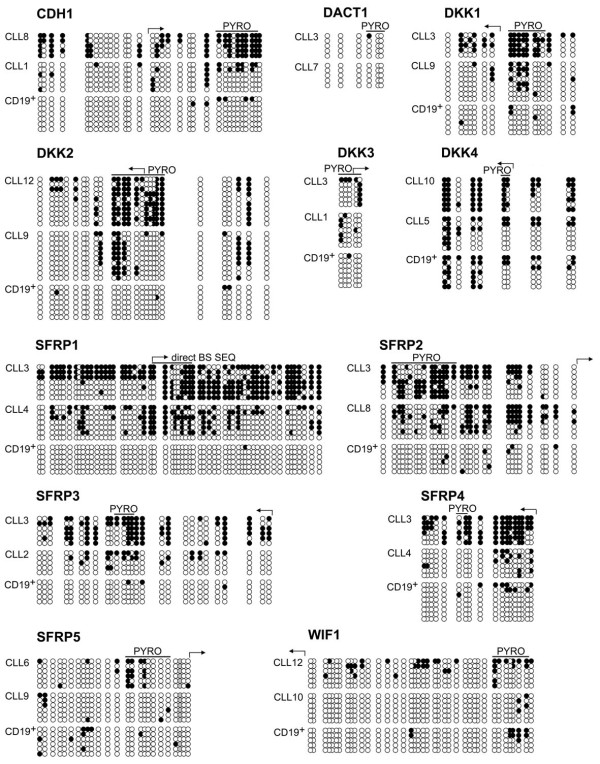
**Results of Sanger bisulphite sequencing of patient and control samples. **For each gene, DNA of the patient with the maximal methylation within the group of twelve (top) and one with an average degree of methylation (middle) were analysed. Also the result of one randomly selected control (CD19^+^) is shown (bottom). Every row of circles represents the CpG sites of an individual clone. The density of the CpG sites in the genomic sequence is represented by the distances between the circles. Open and filled circles stand for unmethylated and methylated CpG sites, respectively. Grey circles denote point mutations found in *SFRP5 *(CLL6, CLL9, CD19^+^). Horizontal lines above the circles labelled with “PYRO” indicate the regions quantified by bisulphite pyrosequencing. Arrows indicate the positions of known or presumed transcriptional start sites.

To exclude a possible contribution of additional covariates such as country of origin, sex or age of the individuals to the observed alterations of DNA methylation, we took them into account by using generalised least squares models. A disease-specific nature of methylation differences between compared groups was confirmed for all the genes, which exhibited differential methylation between the groups [see Additional file 2: Table S 2]. The results obtained from bisulphite pyrosequencing were validated by genomic bisulphite Sanger sequencing of randomly cloned PCR-products (Figure 3). The data obtained were in full agreement with the results of the pyrosequencing assay. In combination, the sequencing results indicate that the genes of most Wnt/β-catenin pathway inhibitors are prone to aberrantly high methylation in CLL.

### Expression of the wnt/β-catenin pathway inhibitors is associated with the methylation status

**Figure 4:**
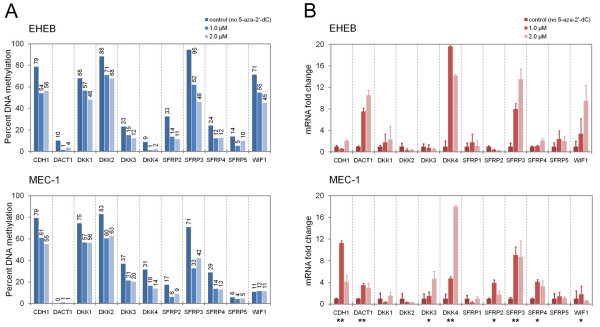
**Quantification of methylation degrees and expression of the inhibitor genes in CLL cell lines EHEB and MEC-1 upon treatment with 5-aza-dC. **(**A**) The average percentage of methylation was recorded by bisulphite pyrosequencing after 72 h growth in pure medium (deep blue bars) and in presence of 1.0 or 2.0 μM 5-aza-dC (intermediate and light blue bars), respectively. (**B**) Quantification of mRNA expression levels by PCR. As in panel A, control and experimental conditions are represented by different shades of red. Three separate measurements were performed for each sample. *GAPDH *was used as an internal control. The expression in untreated cells was set to 1. Significant induction of mRNA expression in both or one cell line is indicated with ** or * at the bottom.

In order to ascertain the role of methylation in the regulation of expression, mRNA levels of the twelve genes were analysed by quantitative PCR in the cell lines EHEB and MEC-1 after treatment with 5-aza-2´deoxycytidine (5-aza-dC). Incorporation of this nucleoside analogue into DNA is known to capture covalently DNA methyltransferases, thereby inducing DNA hypomethylation [44]. At a concentration of 1.0 and 2.0 μM, the drug induced on average a 25% decrease in the methylation level of the strongly methylated genes (Figure 4A). This decrease in DNA methylation was associated with a significant transcriptional re-activation of *CDH1DKK3DKK4SFRP2SFRP3SFRP4* and *WIF1* in at least one of the two cell lines (Figure 4B ).

It is interesting to note that the expression of *SFRP3* was induced 13.5- and 9.1-fold in EHEB and MEC-1, although no CpG island has been annotated from the genomic sequence [16]. This fact may highlight the rather artificial character of CpG island definition. The effect of the methylation decrease on expression was striking, which may actually be particularly due to the small number of CpG sites in the region (although some indirect activation mechanism cannot be excluded either). *DKK1DKK2* and *SFRP1* were also highly methylated in both cell lines. Still, treatment with 5-aza-dC did not result in a significant induction of expression, although methylation was reduced to a degree that was similar to that of *SFRP3*. The genes *DKK1DKK2* and *SFRP1* do have CpG islands in their sequences. Apparently, the demethylation effect was too little or insufficient on its own to change the overall blockage of expression.

Expression of *DACT1* exhibited yet another, very contrasting result. It was substantially up-regulated after incubation with 5-aza-dC (10.5-fold and 3.5-fold in EHEB and MEC-1, respectively), even though the gene was essentially unmethylated even prior to the treatment. This clearly indicates an indirect control of this gene’s activity by the variation of the degree of DNA methylation; its own sequence is not affected, however.

### Upon 5-aza-dC treatment, CLL cell lines accumulate β-catenin that binds to the induced E-cadherin

**Figure 5:**
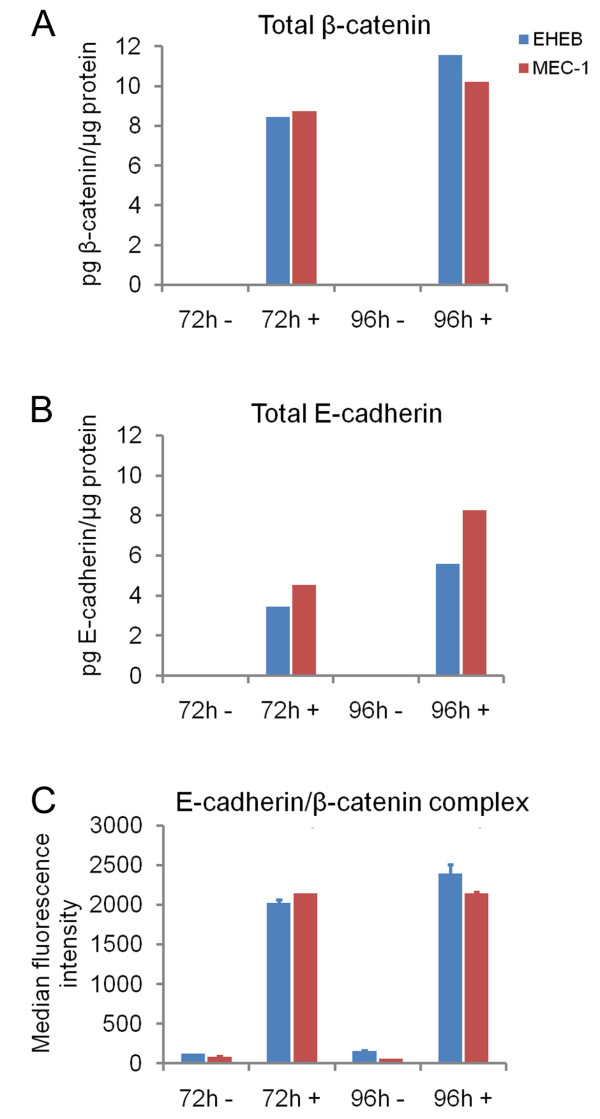
**Quantification of the amounts of β-catenin, E-cadherin and the E-cadherin/β-catenin complex in the cell lines EHEB (blue bars) and MEC-1 (red bars). **The amount of β-catenin (**A**), E-cadherin (**B**) or the E-cadherin/β-catenin complex (**C**) was determined after 72 h and 96 h cell growth in presence (+) or absence (−) of 2.0 μM 5-aza-dC. The measurements without 5-aza-dC shown in panels (**A**) and (**B**) produced only residual signal intensities.

In order to demonstrate the functional relevance of epigenetic control of antagonists to the overall regulation of the Wnt/β-catenin pathway, we looked at the variation in the amounts of transcriptionally active (non-phosphorylated) β-catenin and E-cadherin in EHEB and MEC-1 cell lines. The amount of non-phosphorylated β-catenin correlates directly with the activity of the pathway. For this analysis, we took advantage of suspension bead arrays, which had already been used for the detection of different cytoplasmic fractions of β-catenin [41]. No constitutive Wnt/β-catenin signalling could be detected in the cell lines. The non-phosphorylated fraction of β-catenin was hardly detectable (data not shown) at either condition, suggesting dormancy of this pathway route or a very efficient degradation process. However, the β-catenin level strongly increased in cell lysates after a treatment of the cells with 2.0 μM 5-aza-dC for 72 h and 96 h (Figure 5A), indicating a methylation controlled silencing in cell lines. Concomitantly to the increase in β-catenin levels, also the amount of E-cadherin increased substantially upon *CDH1* hypomethylation, while the protein was basically undetectable under control conditions (Figure 5A). As expected, β-catenin and E-cadherin formed a complex, which as a result of the higher expression levels of *CDH1* was also present at much higher concentrations after treatment with 5-aza-dC (Figure 5C).

## Discussion

Aberrant activation of the Wnt/β-catenin pathway in primary CLL is known to contribute to the defect in apoptosis inherent to this malignancy [3,6,7,17]. Different molecular mechanisms for this were suggested, including the autocrine activation by the overexpressed Wnt ligands [7], silencing of E-cadherin due to aberrant splicing [45] or epigenetic down-regulation of pathway inhibitors by promoter hypermethylation [16]. Stimulated by the findings of aberrantly methylated *CDH1*[19] and *SFRP*s [16] as well as our own evidence on hypermethylation of *DKK2* and *DKK3* in primary CLL, we quantitatively characterised the methylation status and the expression of the Wnt/β-catenin pathway inhibitors. Ten of the twelve genes exhibited high DNA methylation in at least one of the CLL cell lines and also in patient samples compared to non-tumour material. The absolute level of methylation, however, varied strongly between the genes.

By far the biggest difference in methylation between tumour and normal could be seen for *SFRP1*, which was hypermethylated in all tumour samples. Surprisingly, however, the apparent change in transcript level upon DNA demethylation with 2 μM 5-aza-dC was insignificant. In contrast, methylation in the promoter regions of *DACT1* and *DKK4* did not differ between tumour and normal. However, their transcription was affected by changes to the degree of DNA methylation, although for *DACT1* this happened clearly indirectly via a yet unknown factor. The fact that DNA methylation was found to be involved in the regulation of the antagonist genes’ expression in the cell lines and the close similarity of the methylation patterns to those of primary CLL B cells suggests the existence of an epigenetic silencing mechanism in this haematological malignancy.

Owing to the absence of the CpG island within its 5´ region, *SFRP3* was the only member of the *SFRP* class, whose methylation status had not been analysed in most of reports published earlier, irrespective of the studied tumour type. However, we could demonstrate that hypermethylation of CpG sites in the first exon was associated with an apparent transcriptional down-regulation to an extent that was well beyond that seen for other genes. Also overall, the variation of methylation upon addition of the demethylation agent differed between genes. There might be a correlation between the intensity of the effect observed and the mere number of CpG sites or their frequency in a given stretch of DNA sequence. However, the data set from this study was too small for any significant evaluation and more quantitative analyses are required to proof an actual relationship.

Also, the degree of demethylation induced by 5-aza-dC had different apparent effects on the transcription levels. The extent of variation may be controlled by the sequences next to the CpG sites. Such an effect is known for the formation of left-helical Z-DNA structures, which are most likely to occur in methylated d(CG) sequences [46]. A conformational twist from right- to left-helical secondary structure can occur from one base to the next and back, resulting in a net structural variation that only disturbs or relaxes the right-turning helix rather than inducing a strong conformational change. This variation in the structural components of a sequence could explain an associated variation in gene activity and could topologically affect also DNA stretches that have some distance from the actual CpG site.

Because epigenetic down-regulation of the Wnt/β-catenin pathway inhibitors may be instrumental in the constitutive Wnt/β-catenin signalling in primary CLL [3,6,7,17], we wondered if the pathway is active in the CLL cell lines and can be modulated upon DNA hypomethylation. However, no constitutive Wnt/β-catenin signalling could be detected in either of the two cell lines by measuring the level of the transcriptionally active β-catenin fraction. This obvious discrepancy with primary CLL [3,7] might reflect a secondary loss of the constitutively active pathway in the established cell lines. This observation is in agreement with a recent report [6], which has documented much less β-catenin and lymphoid enhancer-binding factor 1 (LEF-1, a direct target of the pathway [47]) in the CLL cell lines JVM-1 and MEC-1 in comparison with the patient samples. Given a dormant Wnt/β-catenin pathway in the CLL cell line models, it was therefore not possible to ascertain, if pharmacological restoration of all the pathway antagonists can affect its activity. Nevertheless, the role of epigenetic silencing of one of the inhibitors, E-cadherin, could be demonstrated. We showed that its expression is regulated by promoter methylation both on transcriptional and protein levels. Significantly up-regulated upon 5-aza-dC treatment, E-cadherin binds β-catenin thereby capturing it on the cellular membrane. We speculate that epigenetic restoration of E-cadherin expression in the cells with aberrantly active Wnt/β-catenin signalling might suppress it in a similar way as the enforced E-cadherin expression in the primary CLL B cells lead to down-regulation of the pathway [45]. Thus, this finding may be of therapeutic interest.

Finally, observations from this study draw attention to two aspects, which warrant further clarification. First, the concurrent hypermethylation and silencing was shown for the genes of a single pathway, which are located on different chromosomes. This finding may support a model of carcinogenesis suggested earlier, in which epigenetic silencing is not completely stochastic but might reflect the existence of a directed program, by which functionally related groups of genes important for development of tumours are silenced through promoter methylation [48]. Our data may add further evidence for such a guiding role of Polycomb-group repressive complexes (PRC) in patterning aberrant DNA hypermethylation in cancer. These epigenetic regulatory proteins induce repressive chromatin states by covalently modifying histones (H3K27me3) within promoters of many developmentally regulated genes in embryonic and adult stem cells [49,50]. Also in B cells, a PRC2 component EZH2 was shown to contribute to epigenetic reprogramming of naïve B cells as they transit through germinal centre, which may facilitate proliferation and lymphomagenesis [51]. Given the facts that (1) promoter regions of the Wnt pathway antagonists are repressively marked with H3K27me3 in either naïve B cells, centroblasts or embryonic stem cells [51,52], (2) promoters of *DKK1, DKK2, SFRP1* and *SFRP2* are occupied with the PRC2 component EZH2 in these cells, (3) recruitment of DNA methyltransferases by EZH2 [53] and (4) age dependent aberrant hypermethylation of PRC targets [54] have been reported, it is plausible to assume that concurrent hypermethylation of specific groups of genes is an interrelated part of general epigenome reprogramming orchestrated by PRC.

Second, despite the fact that cancer-associated CpG hypermethylation has been shown early in development of solid tumours [55], nothing is known about epigenetic alterations in a CLL precursor state monoclonal B cell lymphocytosis (MBL) [56]. However, up-regulation of LEF-1, a direct target of the Wnt pathway and a pro-survival factor, has recently been reported in MBL patients, who are known to be at risk for progression to CLL [3]. Therefore, deregulation of the Wnt/β-catenin pathway may have a role in CLL leukaemogenesis and further methylation analysis of the antagonists in MBL is desirable in view of possible benefits for CLL prevention by DNA demethylating drugs, if epigenetic aberration of these genes is detected at an early stage of MBL.

## Conclusions

Our results show concurrent hypermethylation of multiple Wnt/β-catenin pathway inhibitor genes in CLL. The methylation status is associated with expression, what suggests epigenetic silencing mechanism of this signalling route. Aberrant hypermethylation of the whole group of functionally related genes may not be completely stochastic but result from the epigenome reprogramming orchestrated by Polycomb-group repressive complexes. The data are of interest in the context of epigenetic-based therapy.

## Competing interests

The authors declare that they have sno competing interests.

## Authors’ contributions

EAM, KL, OP, TOJ, JDH conceived and designed the experiments. EAM, KL, AS, MS performed the experiments. EAM, SEM, KL, AS analysed the data. IAV, KDK, OBK, AAG provided patient samples and clinical data. EAM, JDH, SEM wrote the manuscript. All authors read and approved the final version of the manuscript.

## Pre-publication history

The pre-publication history for this paper can be accessed here:

http://www.biomedcentral.com/1471-2407/12/213/prepub

## Supplementary Material

Additional file 1 **Table S1. **Demographic and clinical characteristics of CLL patients and healthy donors. Click here for file

Additional file 2 **Table S2. **The optimal GLS models found for each of the examined genes by a stepwise backward reduction of the full model described in Material and Methods. Click here for file
