# Supplementary material for: Concurrent epigenetic silencing of wnt/β-catenin pathway inhibitor genes in B cell chronic lymphocytic leukaemia
Source: BMC Cancer. 2012 Jun 6;12:213. doi: 10.1186/1471-2407-12-213 (PMC3489542; doi:10.1186/1471-2407-12-213)
Supplement: Additional file 1 — Table S1. Demographic and clinical characteristics of CLL patients and healthy donors. [file 1471-2407-12-213-S1.pdf]

Additional file 1

Demographic and clinical characteristics of CLL patients and healthy donors

Status	Sample ID	Age	Sex	Country of origin	Staging		Leukocytosis, x10^9/L	Lymphocyte gate, %	$CD19^+$ in lymphocyte gate, %	$CD19^+/CD5^+$, % of total $CD19^+$	T cells in lymphocyte gate, %
					Rai	Binet					
CLL	CLL1	74	M	Russia	2	B	350.0	75.0	88.0	96.7	8.1
	CLL2	58	F	Russia	0	A	22.9	46.7	77.0	92.8	20.5
	CLL3	83	F	Russia	4	C	138.0	66.0	79.0	68.0	15.8
	CLL4	64	M	Russia	4	C	62.4	68.6	95.0	83.7	6.8
	CLL5	55	F	Russia	0	A	13.4	21.0	45.0	57.7	53.8
	CLL6	67	F	Russia	0	A	11.3	83.0	77.0	92.0	20.9
	CLL7	66	F	Russia	0	A	20.6	92.0	75.0	84.4	26.3
	CLL8	67	F	Russia	0	A	17.8	14.0	16.0	15.0	73.5
	CLL9	68	M	Russia	4	C	12.6	78.0	97.0	92.0	4.9
	CLL10	42	F	Russia	1	A	25.2	68.0	98.0	89.0	0.9
	CLL11	51	F	Russia	0	A	12.7	94.0	98.0	93.0	0.8
	CLL12	62	F	Russia	2	A	140.0	96.0	86.0	85.0	12.7
$CD19^+$ control	$CD19^+$(1)	29	M	Russia	-	-	-	-	-	-	-
	$CD19^+$(2)	50	F	Germany	-	-	-	-	-	-	-
	$CD19^+$(3)	41	F	Germany	-	-	-	-	-	-	-
	$CD19^+$(4)	24	M	Germany	-	-	-	-	-	-	-
	$CD19^+$(5)	19	F	Germany	-	-	-	-	-	-	-
