# Supplementary material for: Concurrent epigenetic silencing of wnt/β-catenin pathway inhibitor genes in B cell chronic lymphocytic leukaemia
Source: BMC Cancer. 2012 Jun 6;12:213. doi: 10.1186/1471-2407-12-213 (PMC3489542; doi:10.1186/1471-2407-12-213)
Supplement: Additional file 2 — Table S2. The optimal GLS models found for each of the examined genes by a stepwise backward reduction of the full model described in Material and Methods. [file 1471-2407-12-213-S2.pdf]

Additional file 2.

The optimal GLS models found for each of the examined genes by a stepwise backward reduction of the full model described in Material and Methods

Gene	Variables left in the model	Intercept (significance)	Coefficient 1 (significance)	Coefficient 2 (significance)
CDH1	Status + Age	1.587 (P < 0.001)	3.043 (P < 0.001)	0.042 (P = 0.025)
DACT1	Status	0.405 (P < 0.001)	1.020 (P = 0.005)	n.a.
DKK1	Status	2.250 (P < 0.001)	3.326 (P < 0.001)	n.a.
DKK2	Status	2.661 (P < 0.001)	2.987 (P < 0.001)	n.a.
DKK3	Status	2.307 (P < 0.001)	1.730 (P < 0.001)	n.a.
DKK4	none	n.a.	n.a.	n.a.
SFRP2	Status	2.738 (P < 0.001)	1.445 (P = 0.023)	n.a.
SFRP3	Status	2.436 (P < 0.001)	2.453 (P < 0.001)	n.a.
SFRP4	none	n.a.	n.a.	n.a.
SFRP5	Sex	3.778 (P < 0.001)	-1.082 (P = 0.016)	n.a.
WIF1	Status + Sex	3.770 (P < 0.001)	1.699 (P = 0.004)	-1.070 (P = 0.022)

Note: Coefficient 1 and coefficient 2 correspond to the coefficients of the first and second variables in each model, respectively. Significance of the model parameters was estimated using the analysis of variance (Pinheiro and Bates, 2000). Therefore, the reported P-values refer to those produced by F-tests. In each of the significant models, the variance of residuals was allowed to differ between the groups of healthy and diseases individuals (except for the model of SFRP5, in which the variance was different among sexes).
The abbreviation “n.a.” stands for “not applicable”.

References

Pinheiro JC, Bates DM: *Mixed-Effects Models in S and S-PLUS*. New York: *Springer-Verlag*; 2000.

Pinheiro J, Bates D, DebRoy S, Sarkar D and the R Development Core Team. nlme: Linear and Nonlinear Mixed Effects Models. R package version 3.1-102. [http://cran.r-project.org/web/packages/nlme]

R Development Core Team: R: A language and environment for statistical computing. R Foundation for Statistical Computing. [http://www.R-project.org]
